# Evidence for a Continuous Drift of the HIV-1 Species towards Higher Resistance to Neutralizing Antibodies over the Course of the Epidemic

**DOI:** 10.1371/journal.ppat.1003477

**Published:** 2013-07-04

**Authors:** Mélanie Bouvin-Pley, Marion Morgand, Alain Moreau, Pauline Jestin, Claire Simonnet, Laurent Tran, Cécile Goujard, Laurence Meyer, Francis Barin, Martine Braibant

**Affiliations:** 1 Université François Rabelais, Inserm U966, Tours, France; 2 Université Paris Sud, CESP Inserm U1018, Paris, France; 3 AP-HP Hôpital de Bicêtre, Le Kremlin-Bicêtre, France; 4 for the ANRS SEROCO and PRIMO study groups; 5 Centre National de Référence VIH, Laboratoire de Bactériologie-Virologie, CHU Bretonneau, Tours, France; University of Zurich, Switzerland

## Abstract

We compared the neutralization sensitivity of early/transmitted HIV-1 variants from patients infected by subtype B viruses at 3 periods of the epidemic (1987–1991, 1996–2000, 2006–2010). Infectious pseudotyped viruses expressing envelope glycoproteins representative of the viral quasi-species infecting each patient were tested for sensitivity to neutralization by pools of sera from HIV-1 chronically infected patients and by an updated panel of 13 human monoclonal neutralizing antibodies (HuMoNAbs). A progressive significantly enhanced resistance to neutralization was observed over calendar time, by both human sera and most of the HuMoNAbs tested (b12, VRC01, VRC03, NIH45-46^G54W^, PG9, PG16, PGT121, PGT128, PGT145). Despite this evolution, a combination of two HuMoNAbs (NIH45-46^G54W^ and PGT128) still would efficiently neutralize the most contemporary transmitted variants. In addition, we observed a significant reduction of the heterologous neutralizing activity of sera from individuals infected most recently (2003–2007) compared to patients infected earlier (1987–1991), suggesting that the increasing resistance of the HIV species to neutralization over time coincided with a decreased immunogenicity. These data provide evidence for an ongoing adaptation of the HIV-1 species to the humoral immunity of the human population, which may add an additional obstacle to the design of an efficient HIV-1 vaccine.

## Author Summary

Most of the patients develop autologous neutralizing antibodies (NAbs) during HIV-1 infection. These NAbs drive the viral evolution and lead to the selection of escape variants at the individual level. The aim of our study was to check if, subsequently to the selective pressure exerted by the individual NAbs responses, the HIV-1 species has evolved at the population level towards an enhanced resistance to antibody neutralization. By comparing HIV-1 subtype B variants collected at three periods spanning more than 2 decades, we found a significantly progressive enhanced resistance to neutralization of the HIV-1 species over time. In addition, the enhanced resistance of the HIV species to neutralization coincided with a decreased capability of the virus to induce NAbs in infected patients. Despite this evolution, one combination of two human monoclonal broadly NAbs still were able to neutralize the most recent HIV-1 variants, suggesting that this combination should be preferentially included in future human immunoprophylaxis trials.

## Introduction

Thirty years after the discovery of the human immunodeficiency virus (HIV), the development of an effective vaccine remains an elusive goal. Experiments of passive immunization and vectored immunoprophylaxis in animal models have shown that human monoclonal (HuMo) broadly neutralizing antibodies (NAbs) can fully protect against HIV-1 infection [1]–[11]. However the design of an immunogen able to induce NAbs that would mediate potent cross-clade HIV-1 neutralization has not been reached so far. The identification of antibody specificities able to neutralize most of the currently circulating HIV-1 variants remains therefore a major focus of vaccine design.

During natural HIV-1 infection, most of the patients develop autologous NAbs at the early stage of infection [12]–[17]. NAbs are directed against the gp120 and gp41 subunits of the viral envelope glycoprotein (Env). The breadth of the autologous response is relatively narrow, as illustrated by its inability to neutralize heterologous isolates [12], [18]–[20] and the absence or low level of protective activity against superinfection [21]–[23]. These antibodies do not seem to protect against disease progression but exert a selective pressure that drives the viral evolution and leads to the rapid selection of escape Env variants [12], [13], [24]–[26]. The molecular basis of HIV-1 escape to autologous neutralization involves multiple mechanisms, including single amino acids substitutions, insertions/deletions in the variable regions of the gp120 and an increased number and/or changing positions of potential N-linked glycosylation sites (PNGS) at its surface [13], [20], [24], [27], [28]. Nevertheless, it has become clear that a substantial number of HIV-1 infected individuals develop NAbs after 2 or 3 years of infection able to neutralize efficiently heterologous primary isolates of various subtypes [29]–[32]. This means that the relevant epitope(s) exist toward which a specific response can be mounted, at least in some individuals. Prior to 2009, only four HuMo broadly Nabs, i.e. b12, 2G12, 2F5 and 4E10, had been isolated from such individuals [33]–[37]. Recently, a “second generation” of HuMoNAbs (particularly the PG, PGT and VRC series) that are 10 to 100-fold more potent than the first generation HuMoNAbs were identified [38]–[41]. Several studies suggested that broad and potent neutralizing activity in most of the sera from patients with broadly NAbs arises through a limited number of specificities that correspond to the targets of these HuMoNAbs [42]–[45]. These targets are epitopes located within the surface glycoprotein gp120. Some of them overlap the CD4 binding site [39], [46], [47] and others are more complex, of glycopeptidic nature, composed of conserved glycans and amino-acid residues of the V1, V2 and V3 loops [48], [49].

Two years ago, Bunnik *et al* suggested that HIV-1 might be evolving at the population level towards an enhanced resistance to antibody neutralization, subsequently to the selective pressure exerted by the individual NAbs responses [50]. Comparing HIV-1 variants isolated from patients of the Amsterdam Cohort Studies either early in the epidemic (1985–1989) or more recently (2003–2006), they found an enhanced neutralization resistance of HIV-1 during the course of the epidemic, especially towards HuMoNAbs targeting epitopes of the CD4-binding site [50], [51]. This finding may have major consequences for vaccine development. Therefore, we conducted the present study in order to both validate and extend the comprehension of the phenomenon. We compared the neutralization sensitivity of HIV-1 subtype B early/transmitted variants issued from French individuals at 3 periods of the epidemic, spanning more than 20 years (1987–1991/1996–2000/2006–2010). Their neutralization sensitivity was tested using both polyclonal sera from HIV-1 infected patients and a large and updated panel of 13 HuMoNAbs, including the most efficient NAbs among those described to date. Our results confirm a clear continuous and progressive enhanced resistance to neutralization over time, providing evidence for an ongoing adaptation of the HIV-1 species to the humoral immunity of the human hosts over the course of the epidemic. However and despite this evolution, we found that one combination of two HuMoNAbs still should neutralize the most recently circulating HIV-1 variants, even at a relatively low concentration (≤1 µg/mL). Therefore, in addition to bring a basic knowledge on the interplay between HIV and the human species, our data provide a rationale for the selection of the HuMoNAbs that should be preferentially used for HIV immunoprophylaxis, especially for the emerging strategy of antibody gene transfer [11].

## Results

### Increasing resistance of early/transmitted HIV-1 variants to neutralizing antibodies over the course of the epidemic

The HIV-1 population that we studied was isolated from 40 patients enrolled at time of primary infection in the French ANRS PRIMO and SEROCO cohorts at three periods of the epidemic: between 1987 and 1991 (Historical patients, HP), 1996 and 2000 (Intermediate patients, IP) and 2006 and 2010 (Contemporary patients, CP). They were carefully selected to be comparable for each period in the following way: all patients were Caucasian men having sex with men (MSM), infected by clade B viruses. They had similar distribution of viral loads (median value: 5.0, 5.1 and 5.2 log_10_ copies/mL for HP, IP and CP, respectively) and similar distribution of CD4 T-cell counts (median value: 507, 619 and 571 cells/mm^3^ for HP, IP and CP, respectively) (Table S1) at time of sample collection. In order to limit both the viral diversity of the quasi-species infecting each patient and the influence of the development of an autologous humoral immune response on the neutralization sensitivity of viruses, blood samples were collected shortly after infection (before 3 months post-infection except for a few cases) (Table S1). The variants that we analyzed were therefore considered as early/transmitted viruses.

Pseudotyped viruses expressing envelope glycoproteins (Env) variants representative of the viral quasi-species infecting each patient were generated from the entire *env* gene amplified by RT-PCR from plasma viral RNA. Infectious pseudoviruses were obtained for 11 to 15 patients infected at each of the 3 periods. They were compared for their sensitivity to neutralization by pools of sera from chronically infected patients infected early in the epidemic (1987–1991) or more recently (2003–2007) (Table S2), and by a panel of 13 HuMoNabs targeting major neutralizing epitopes. These included b12, VRC01, VRC03 and NIH45-46^G54W^, an engineered mutant derived from a clonal variant of VRC01, which target the CD4-binding site of gp120 [39], [40], [46], [47]; PG9, PG16 and PGT145 which recognize a glycan-dependant quaternary epitope in the gp120 variable loops 1 and 2 (V1/V2) [38], [41], [48], [49]; PGT121, PGT128, PGT135 and 2G12 which target gp120 glycan-dependant epitopes within or near the variable loop 3 (V3) of gp120 [35], [41], [49], [52]; and 2F5 and 4E10 which target the membrane-proximal external region (MPER) of gp41 [37], [38], [53], [54]. The sensitivity to neutralization was measured using a single round of infection in TZM-bl target cells. The 50% inhibitory concentrations (IC_50_) were determined for each pool of sera (reciprocal of dilution) and for each HuMoNab (antibody concentration).

**Figure 1:**
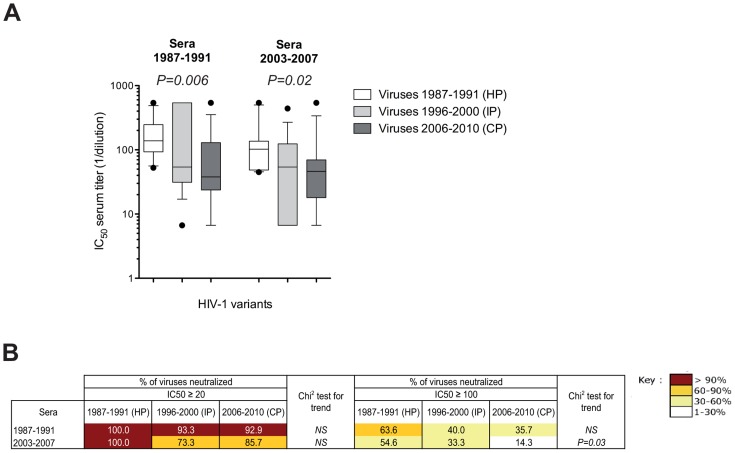
Enhanced resistance of clade B early/transmitted HIV-1 variants to neutralization by polyclonal sera over the course of the epidemic. [A] Comparison of the neutralization sensitivity of Env-pseudotyped viruses derived from historical patients (HP, n = 11), intermediate patients (IP, n = 15) and contemporary patients (CP, n = 14) by pools of sera from chronically infected patients infected early in the epidemic (1987–1991) or more recently (2003–2007). Box plots show the distribution of antibody titers (IC_50_) in each pool of sera towards pseudotyped viruses of each period; the horizontal lines represent the 10^th^, median and 90^th^ percentiles. Each data represents the mean value of the assay performed in duplicate. Differences of neutralization sensitivity between viruses over calendar time were evaluated using a Jonckheere-Terpstra test. [B] Comparison of the neutralization coverage of the two pools of sera (1987–1991 and 2003–2007) at two concentrations (IC_50_≥20 or IC_50_≥100) towards pseudotyped viruses from HP, IP and CP. Differences of neutralization coverage of viruses from HP to CP were evaluated using a Chi^2^ test for trend.

We observed a significantly progressive decrease over time in sensitivity to neutralization of the viruses by the two pools of sera (1987–1991 sera, median IC_50_: 138.3, 54.0 and 37.8 for HP, IP and CP, respectively, *P* trend = *0.006*; 2003–2007 sera, median IC_50_: 102.2, 54.0 and 46.2 for HP, IP and CP, respectively, *P* trend = *0.02*) (Fig. 1 A and Table S3). At high concentrations (IC_50_≥20), the neutralization coverage (percentage of neutralized viruses) by the pools of sera was high (range: 73.3% to 100%) (Fig. 1 B). In contrast, when higher stringent conditions were considered (IC_50_≥100), a significant and progressive decrease in the percentage of neutralized viruses was observed over time for the 2003–2007 pool of sera (*P* trend = *0.03*). Although not statistically significant, a similar trend was observed for the pool of sera from the 1987–1991 period (Fig. 1 B). Taken together, these observations exclude the possibility that the lower sensitivity to neutralization of contemporary HIV variants is related to the calendar time from which the sera originated.

**Figure 2:**
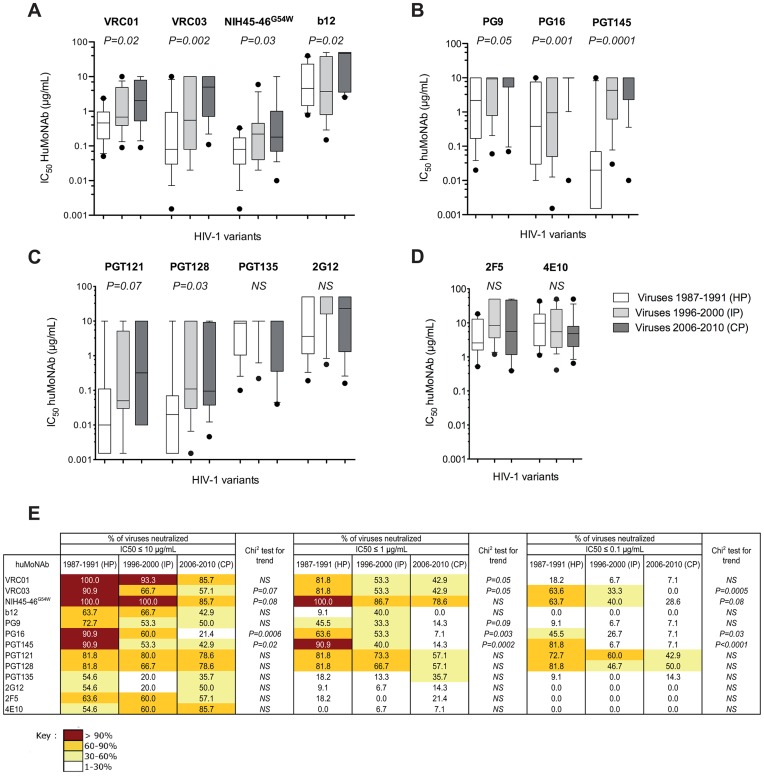
Enhanced resistance of clade B early/transmitted HIV-1 variants to neutralization by HuMoNAbs over the course of the epidemic. [A to E] Comparison of the neutralization sensitivity of Env-pseudotyped viruses derived from historical patients (HP, n = 11), intermediate patients (IP, n = 15) and contemporary patients (CP, n = 14) by b12, VRC01, VRC03 and NIH45-46G54W [A], PG9, PG16 and PGT145 [B], PGT121, PGT128, PGT135 and 2G12 [C] and 2F5 and 4E10 [D]. Box plots show the distribution of antibody titers (IC_50_) of each HuMoNAb towards pseudotyped viruses of each period; the horizontal lines represent the 10^th^, median and 90^th^ percentiles. Each data represents the mean value of the assay performed in duplicate. Differences of neutralization sensitivity between viruses over calendar time were evaluated using a Jonckheere-Terpstra test. [E] Comparison of the neutralization coverage of each HuMoNabs used at various concentrations (IC_50_≤10 µg/mL, ≤1 µg/mL or ≤0.01 µg/mL) towards pseudotyped viruses from HP, IP and CP. Differences of neutralization coverage of viruses from HP to CP were evaluated using a Chi^2^ test for trend.

A decrease in sensitivity to neutralization of the early/transmitted viruses from CP was also observed for VRC01, VRC03, NIH45-46^G54W^ and b12 (Fig. 2 A). The median IC_50_ of VRC01 and VRC03 increased progressively and significantly from 0.46 and 0.08 µg/mL for HP to 2.03 and 5.00 µg/mL for CP, respectively (VRC01: *P* trend = *0.02*, VRC03: *P* trend = *0.002*). For NIH45-46^G54W^, the median IC_50_ increased from 0.08 for HP to 0.22 µg/mL for IP but remained stable for CP (0.18 µg/mL) (*P* trend = *0.03*). Inversely, the median IC_50_ of b12 did not differ between HP (4.58) and IP (3.73) but increased for CP (46.53) (*P* trend = *0.02*). In addition, the neutralization breadth of VRC01, VRC03 and NIH45-46^G54W^ decreased progressively for viruses from HP to CP (Fig. 2 E). This trend was more obvious at concentrations below 1 µg/mL for VRC01 (*P* trend = *0.05*) and less than 0.1 µg/mL for NIH45-46^G54W^ (*P* trend = *0.08*). The neutralization coverage of b12 was low, even for viruses from HP (Fig. 2 E).

HIV-1 variants from CP were also more resistant to PG9 (median IC_50_: 2.18, 9.30 and 10.00 µg/mL for HP, IP and CP respectively, *P* trend = *0.05*), PG16 (median IC_50_: 0.38, 0.95 and 10.00 µg/mL for HP, IP and CP respectively, *P* trend = *0.001*), and PGT145 (median IC_50_: 0.02, 4.31 and 10.00 µg/mL for HP, IP and CP respectively, *P* trend = *0.0001*) (Fig. 2 B). The neutralization breadth of these three HuMoNAbs decreased progressively from HP to CP, even at high concentrations (Fig. 2 E). This evolution was particularly evident for PGT145.

The decrease in sensitivity to neutralization of viruses from CP was also observed for PGT121 and PGT128 (Fig. 2 C). The median IC_50_ of PGT121 increased progressively from HP to CP (median IC_50_: 0.01, 0.05 and 0.32 µg/mL for HP, IP and CP, respectively). This trend was just above the limit of significance (*P* trend = *0.07*). For PGT128, the median IC_50_ increased from 0.02 µg/mL for HP to 0.11 µg/mL for IP but remained stable for CP (0.10 µg/mL) (*P* trend = *0.03*). The percentages of neutralized viruses by these two antibodies remained high for all subjects at high concentrations (IC_50_≤10 µg/mL), but tended to decrease progressively from HP to CP when higher stringent conditions of neutralization were considered (IC_50_≤1 µg/mL). The sensitivity to neutralization by 2G12 or PGT135 was low, even for viruses from HP (Fig. 2 C and 2 E).

The sensitivity to neutralization by 2F5 and 4E10 was much lower and no trend of an increased resistance to these HuMoNAbs was observed (Fig. 2D and 2E).

Altogether, these results showed that HIV-1 has become more resistant to antibody neutralization over the course of the epidemic. This effect was particularly marked for the pools of sera from HIV-1 infected patients and for the HuMoNAbs that target the CD4-binding site and the glycan-dependant epitopes in V1/V2 and V3.

### Neutralization coverage of HIV-1 variants from contemporary patients by HuMoNAbs combinations

**Figure 3:**
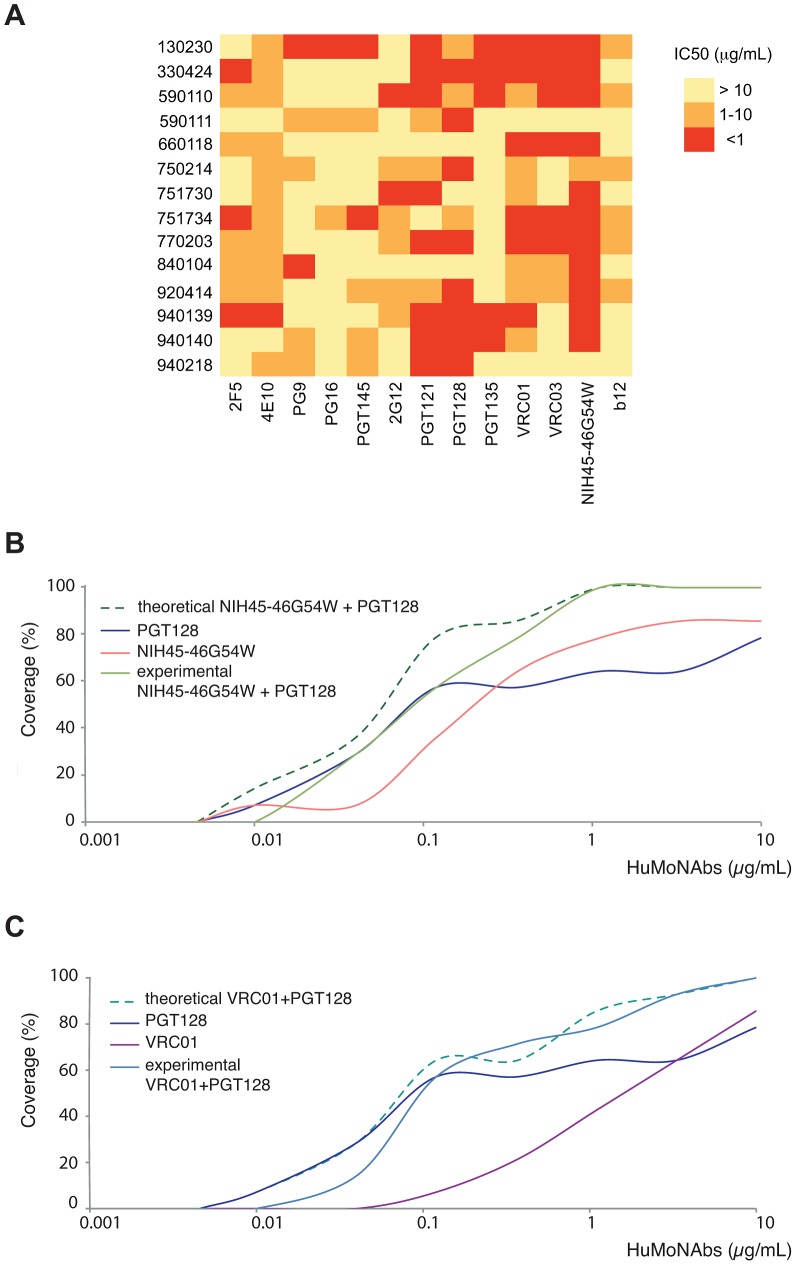
Neutralization coverage of HIV-1 variants from contemporary patients by HuMoNAbs combinations. [A] A heatmap of the neutralizing activities (IC_50_) of the HuMoNAbs against HIV-1 variants from contemporary patients is shown, with increasing darker colors indicating increasing neutralization sensitivity. [B and C] The neutralization coverage of viruses from contemporary patients was tested against various concentrations of a 1∶1 combination of NIH45-45^G54W^ and PGT128 [B] and a 1∶1 combination of VRC01 and PGT128 [C]. Solid lines show the coverage of each HuMoNAb used alone or in combinations. Dashed lines show the theoretical coverage that would be obtained if the neutralizing activities of combined antibodies were fully additive.

Since the HIV-1 early/transmitted viruses from the contemporary patients were the most resistant to neutralization by HuMoNAbs, we tried to predict which antibodies combinations would be the most efficient. The IC_50_ heatmap analysis showed that the engineered antibody NIH45-46^G54W^ was the broadest and most potent antibody (Fig. 3A). It neutralized 12 of 14 viruses, with IC_50_<1 µg/mL for 11 viruses and between 1 and 10 µg/mL for the last one. An identical neutralization breadth was observed for its natural counterpart, VRC01, but at a lower potency, only 6 viruses being neutralized with an IC_50_<1 µg/mL. Interestingly, the two viruses that were resistant to NIH45-46^G54W^ and VRC01 (issued from CP 590111 and 940218) were neutralized by PGT128 efficiently (IC_50_<1 µg/mL), suggesting that combining NIH45-46^G54W^, or to a lesser extend VRC01, with PGT128 would neutralize most or all HIV-1 variants from CP. To test this hypothesis, we investigated the neutralization sensitivity of viruses from CP against a 1∶1 combination of NIH45-46^G54W^ and PGT128, and a 1∶1 combination of VRC01 and PGT128. The observed neutralization coverage by various concentrations of these two combinations was compared to the calculated theoretical coverage that would be obtained if the neutralizing activities were fully additive (Fig. 3 B and C). The experimental data showed that the NIH45-46^G54W^–PGT128 combination was able to neutralize all the viruses with an IC_50_≤1 µg/mL. At this threshold, the VRC01-PGT128 combination neutralized 80% of the viruses. As shown in Fig. 3 B and C, the observed data reached approximatively the theoretical curves, indicating that these two categories of antibodies may counter HIV with an additive effect.

### Molecular characteristics of the early/transmitted viruses included in this study

**Figure 4:**
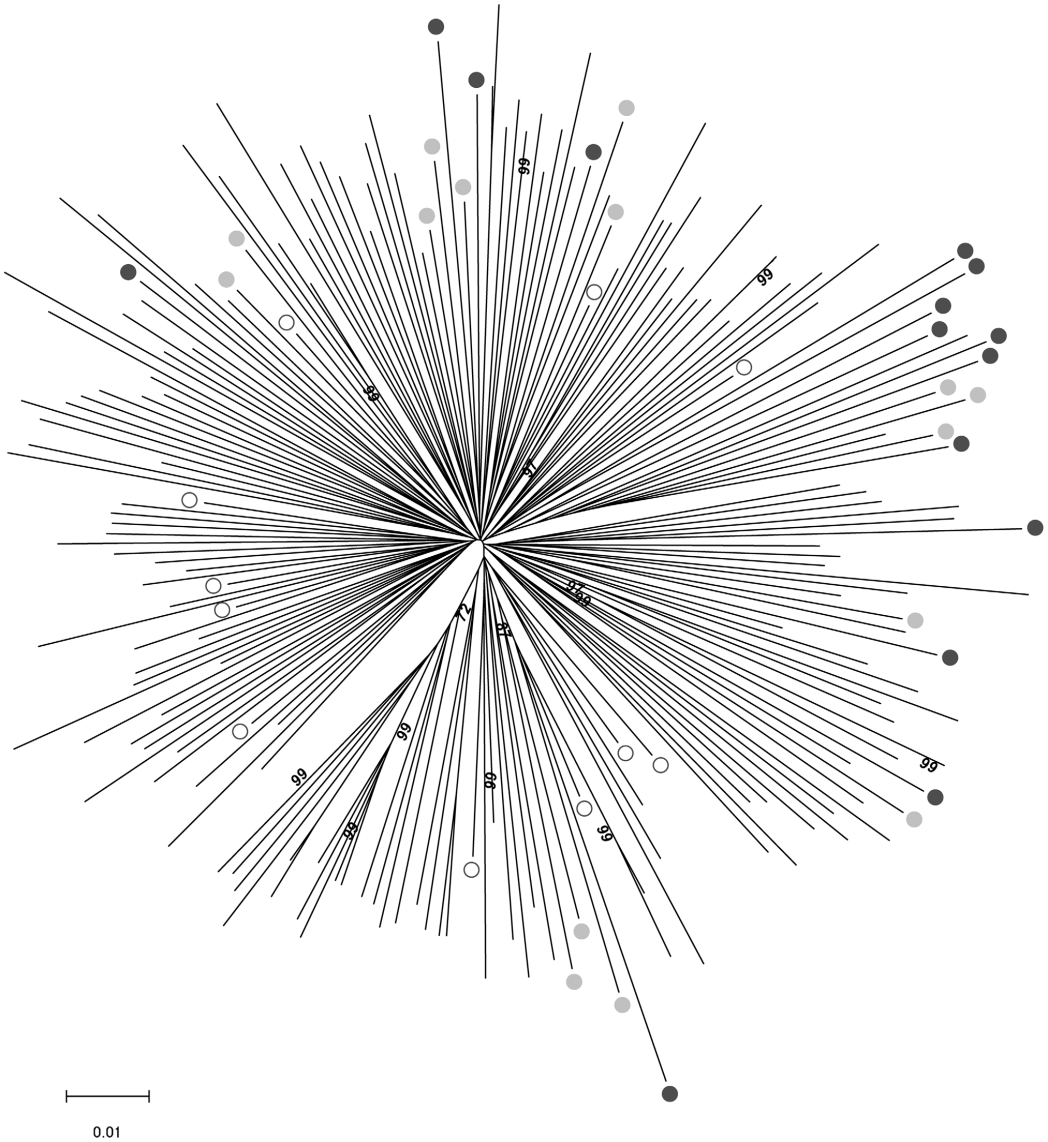
Phylogenetic analysis of full-length*env* sequences. The 40 *env* sequences of the early/transmitted viruses included in our study were aligned with 160 *env* sequences isolated at time of primo-infection from clade B -infected patients, with documented year (1990–2009) and country (25 were from Europe, 113 from USA, 6 from Australia, 15 from Trinidad and 1 from Zambia) of infection. A neighbor-joining tree was constructed using the MEGA software [89]. Open, grey filled and black filled circles identify the sequences derived from the historical, intermediate and contemporary patients of our study, respectively. Bootstrap values above 70% are indicated.

Full-length *env* sequences of the early/transmitted viral population infecting each subject were obtained by direct sequencing from bulk *env* PCR products. These 40 sequences were compared to 160 *env* sequences isolated at the time of primary infection from clade B infected patients with documented year (1990–2009) and country of infection (111 from the United States, 25 from Europe, 6 from Australia, 15 from Trinidad and 1 from Africa). The phylogenetic analysis illustrated in the neighbor joining tree indicated that the *env* sequences of viruses from our study did not cluster in particular branches and were not particularly related to the country of origin or to the study period, historical, intermediate or contemporary (Fig. 4). It suggests that the biological properties that we describe may be representative of the entire clade B HIV-1 population. As it could be expected, the genetic diversity among the *env* sequences increased gradually from HP to CP (mean genetic diversity: 8.8%, 12.4% and 14.8% for HP, IP and CP, respectively). It mirrors the global genetic evolution of HIV-1 over the course of the epidemic [55]–[57], illustrated by the fact that our historical variants are located on shorter branches of the tree when compared to intermediate or contemporary variants (Fig. 4).

**Figure 5:**
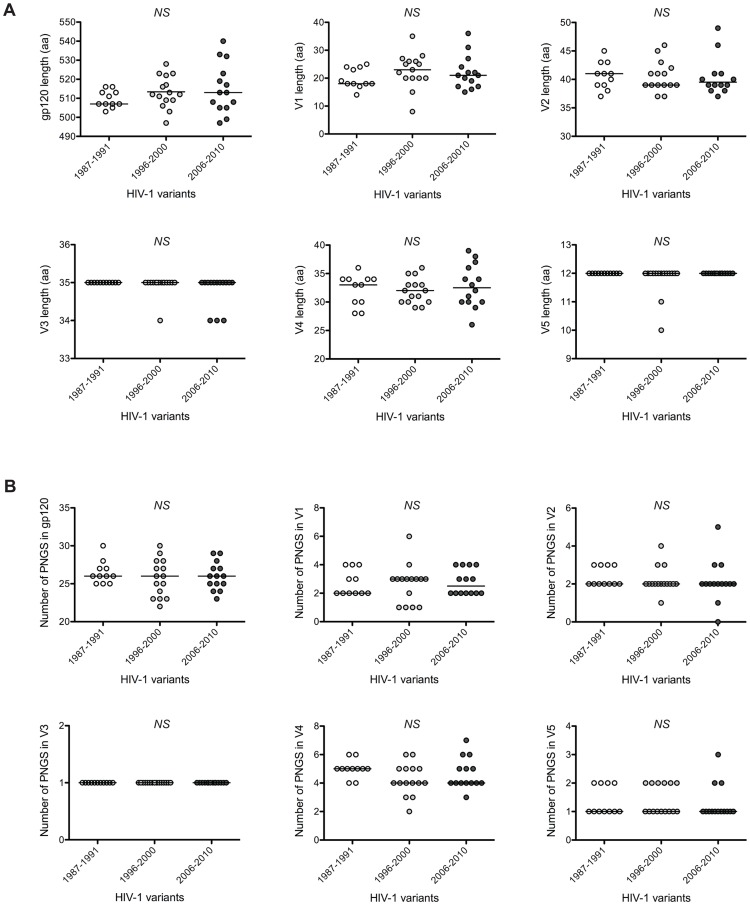
Comparison of length and number of PNGS of gp120 sequences. The lengths [A] and numbers of PNGS [B] of gp120 and of each of its variable loops were compared for early/transmitted viruses from historical patients (HP, n = 11), intermediate patients (IP, n = 15) and contemporary patients (CP, n = 14). Each data point represents the value for one virus. The horizontal line represents the median value. Differences of length or number of PNGS between viruses over calendar time were evaluated using a Jonckheere-Terpstra test.

It has been described regularly that an increase in both length and number of PNGS of gp120, particularly in the variable regions, was associated with the evolution of HIV-1 towards resistance to neutralization at the individual level. We therefore compared these variables between variants issued from HP, IP and CP. No significant differences were observed between the 3 groups of subjects (Fig. 5). We just noticed a slight increase of the global length of gp120 sequences over time (median aa numbers: 507, 512 and 513 for HP, IP and CP, respectively).

**Figure 6:**
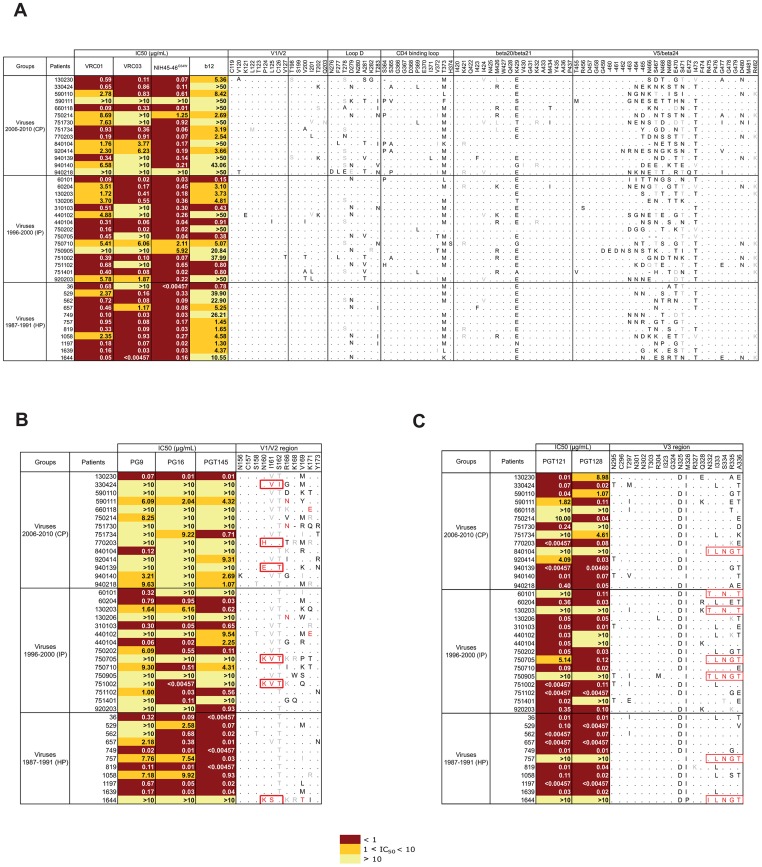
Relationship between resistance to HuMoNAbs and changes in envelope targeted residues. The 40 *env* sequences of viruses from historical (HP), intermediate (IP) and contemporary patients (CP) were aligned with the HXB2 reference sequence. Key residues targeted by b12, VRC01, VRC03 and/or NIH45-46 [A], PGT9, PG16 and/or PGT145 [B], and PGT121 and/or PGT128 [C] are presented. Identical residues are represented by dots. Conserved and non-conserved residues are in grey and black, respectively. Substitutions previously shown to confer neutralization resistance are in red. The neutralization sensitivity (IC_50_) of each HIV-1 variant is indicated for each HuMoNab. IC_50_ values are color coded with increasing darker colors used to indicate increasing neutralization sensitivity.

Because we observed a progressive increase over time in resistance to neutralization by most of the HuMoNAbs, we investigated if changes in key residues targeted by each of these antibodies might be associated with this evolution. Various amino acid substitutions have been identified at sites targeted by VRC01, VRC03, NIH45-46^G54W^ and b12 [39], [40], [46], [47], [58]. We did not find any potential relationship between the resistance of viruses to neutralization by these antibodies and the presence or absence of specific residues at these key positions (Fig. 6 A). In contrast, as previously reported [41], [48], [59], a few amino acids substitutions were found to coincide with a neutralization resistance to PG9, PG16 and PGT145 (Fig. 6 B). These included the loss of a PNGS at position 160 either by the lack of N160 (patients 770203, 940139, 750705, 751002 and 1644) or the absence of the glycosylation sequon despite having N160 (subject 330424); the presence of an Asn at position 166 (patients 590111, 751730 and 130206); the presence of a Thr at position 169 (subject 1644); and the presence of a negatively charged residue (Glu) at position 171 (patients 660118 and 440102). There was a progressive increase in the number of viruses carrying at least one of these substitutions over the course of the epidemic: 1 of 11 (9%), 4 of 15 (27%) and 6 of 14 (43%) for HP, IP and CP (*P = 0.06*, Chi^2^ test for trend). We cannot exclude that other substitutions at these key residues also account for the neutralization resistance of some variants. For instance, the PG9/PG16/PGT145-resistant variant issued from patient 590110 carried three non-homologous substitutions, including the replacement of the positively charged Arg residue at position 166 by a negatively charged Asp residue. In contrast, a few variants were resistant to PG9 (patients 562 and 920203), PG16 (patients 60101 and 920203), or PGT145 (patient 60101), despite having conserved (or similar) residues at key positions required for neutralization sensitivity, suggesting that other molecular determinants are involved in resistance to these HuMoNAbs. Inspection of the V3 region targeted by PGT121 and 128 revealed a shift of the PNGS from N332 to N334 in seven neutralization (or less sensitive)-resistant variants (Fig. 6 C), a modification already reported as involved in sensitivity to PGT121/128 [49], [60]. However, neither the frequency of this change, nor other substitutions in V3 allowed to explain the increasing neutralization resistance to PGT121/128 of HIV-1 variants from CP.

### Decreasing immunogenicity of HIV-1 in terms of NAbs induction

**Figure 7:**
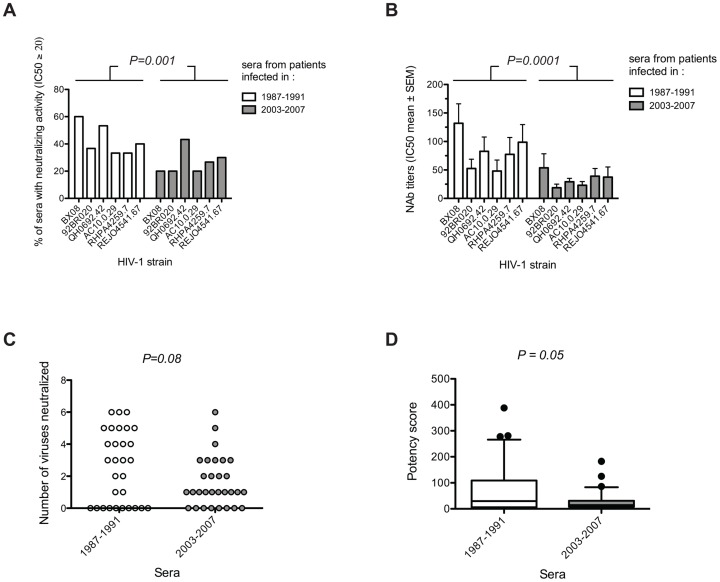
Decreasing immunogenicity of clade B HIV-1 variants in term of NAbs induction over the course of the epidemic. [A and B] Comparison of the frequencies of detection of NAbs [A] and of NAbs titers [B] against 6 heterologous subtype B isolates (BX08, 92BR020, QH0692.42, AC10.0.29, RHPA4259.7 and REJO4541.67) in sera from clade B chronically infected patients at the two extreme periods of the study i.e. 1987–1991 (n = 30) and 2003–2007 (n = 30). Differences in the frequency of detection and the titers of NAbs among sera from the two groups of patients were compared by Chi^2^ or ANOVA tests after adjustment for the strain, respectively. [C] Comparison of the neutralization breadth between the sera of the two periods. Each point represents the value for one serum. [D] Comparison of the potency scores (see methods) between the sera of the two periods. Box plots show the distribution of potency scores of each group of sera; the horizontal lines represent the 10^th^, median and 90^th^ percentiles. Differences between breadth and potency score between the two groups of sera were evaluated using a Wilcoxon signed ranked test.

In order to check whether the evolution of the HIV-1 species towards a higher resistance to neutralization coincided with a poorer capability to induce NAbs, the neutralizing activity of sera from subtype B chronically-infected patients at the two extreme periods of the study, i.e. 1987–1991 (n = 30) and 2003–2007 (n = 30) (Table S2), was tested towards a panel of six heterologous subtype B isolates (Table S4). Sera were collected at least 36 months post-infection from untreated MSM infected by subtype B variants. Although, they were selected to be comparable between the two periods, the median time of collection differed by 4.5 months between the 1987–1991 sera and those of 2003–2007 (43.4 versus 38.9 months, respectively, *P = 0.0006*, Wilcoxon test). However, the time ranges were very similar (34.7 to 51.6 versus 36.2 to 50.9 months post-infection for the 1987–1991 and 2003–2007 sera, respectively) suggesting that the 4.5 months difference in median should not have any impact. These patients had similar viral loads (median: 4.2 and 4.4 log_10_ copies/mL in historical and more recently infected patients, respectively) and similar CD4 T-cell counts (median: 468 and 464 cells/mm^3^ for historical and more recently infected patients, respectively) at the time of sample collection. The panel of heterologous viruses included two clade B primary isolates (BX08 and 92BR020) and four Env-pseudotyped viruses derived from subtype B *env* clones (QH0692.42, AC10.0.29, RHPA4259.7 and REJO4541.67), selected for their moderate (tier 2) sensitivity to neutralization [30], [61], [62]. Highly neutralization-resistant (tier 3) strains were discarded from our analysis due to the scarcity of sera showing any detectable neutralizing activity towards such viruses (data not shown). For each strain, a lower frequency of detection of NAbs (IC_50_ detectable at least at the first serum dilution, i.e. 1∶20) was observed among sera from recently infected patients (2003–2007) compared to those from the earliest period (1997–1991) (Fig. 7 A, Table S4). The differences were statistically significant when considering and adjusting for the strain the entire data set (*P = 0.001*). Similarly, NAbs titers were lower in the serum samples of the recent period when compared to the early period (Fig. 7 B). The differences in Nab titers between the two periods were highly statistically significant when data were analyzed globally (*P = 0.0001*, 2way ANOVA test). We next compared the breadth of the neutralizing response between the two periods, by calculating the number of strains neutralized by each serum at least at the first serum dilution (1∶20). A higher frequency of sera cross-neutralizing at least 4 strains was observed in the earliest period (12/30 *versus* 3/30 sera) whereas most of the sera from the recently infected patients neutralized a limited number (1 to 3) of strains (19/30 *versus* 8/30 sera) (Fig. 7 C). The observed differences were just above the limit of significance (*P = 0.08*). To consider both the breadth and the intensity (measured by the NAb titers) of the neutralizing response, a neutralizing potency score was calculated for each serum (see methods). Lower potency scores were observed for sera of the recent period (median: 14.54) when compared to sera of the early period (median: 29.76; *P = 0.05*, Fig. 7 D). Altogether, these data suggest that patients who were infected more recently developed poorer NAb responses than did those who were infected in the earlier period of the epidemic.

## Discussion

There are strong evidences that HIV-1 evolves considerably faster within hosts than it does at the population level [63], [64]. The preferential transmission of ancestral viruses, stored in long-lived memory CD4+ T cells, may be an important factor contributing to this difference of divergence [65], [66]. However, several studies suggested that there is still an evolution of HIV-1 at the population level, which may have major implications in public health. Results of a recent meta-analysis are consistent with an increased virulence of HIV-1 over the course of the epidemic [67]. Complementary to these analyses and focusing on the sensitivity to NAbs, Bunnik *et al* suggested that HIV-1 was becoming more resistant to the humoral immune response of the host at the populational level [50]. This observation has importance for the fundamental knowledge on host-pathogen interactions, but also may have major consequences for prophylactic antibody-based interventions [68]. In the present study, we validated the reality of the phenomenon and bring detailed descriptive elements. This was made possible through the availability of samples collected at time of primary infection at three different calendar periods of the epidemic, spanning more than 20 years, and the recent availability of a large panel of extremely potent HuMoNAbs [38]–[41]. We found a continuous enhanced resistance of HIV-1 to antibody neutralization over time. This effect was observable for both polyclonal antibodies present in pools of sera from HIV-1 chronically infected patients and most of the HuMoNAbs targeting distinct epitopes of gp120. It included VRC01, VRC03, NIH45-46^G54W^ and b12 which target the CD4-binding site, PG9, PG16 and PGT145 which recognize a glycan-dependant epitope in V1/V2, and PGT121 and PGT128 which target a glycan-dependant epitope in V3. In contrast, no increasing resistance was observed for the gp41-directed HuMoNAbs 2F5 and 4E10. Therefore, our data strengthen the previous results of Bunnik *et al* [50], [51]. Interestingly enough, consistent findings were obtained between these two studies despite the use of a different methodology, a peripheral blood mononuclear cells neutralization assay using primary viruses in the previous study *versus* a TZM-bl assay using pseudotyped viruses in the present study. Both studies focused on the sensitivity to neutralization of early/transmitted viruses, these variants being those selected during primary infection and therefore those that should be preferentially targeted in any prophylactic intervention. In order to limit the bias that could be introduced in such a study, we paid special attention to select samples which were collected as soon as possible after diagnosis of acute infection, or early seroconversion, from a similar transmission group of patients (MSM), with comparable genetic background (Caucasian), and before introduction of any antiretroviral treatment. In addition, our study that covers three periods allows to demonstrate convincingly the dynamic of the enhanced resistance of HIV-1 to the neutralizing response of the host species.

Bunnick *et al* identified an enhanced resistance of HIV-1 to HuMoNAbs targeting the CD4 binding site, b12 and VRC01, and a trend towards enhanced resistance to PG16 [50], [51]. Our study revealed that the enhanced resistance of HIV-1 to neutralization at the population level is not restricted to the CD4-binding site but extends to the conformational glycan-dependant epitopes of the newly described potent HuMoNAbs. This was particularly evident for the HuMoNAbs PG9, PG16 and PGT145 that target the V1/V2 loops, and to a lesser extend for the HuMoNAbs PGT121 and PGT128 that target the V3 loop. The entire set of data suggests that the neutralization escape (or less sensitive) variants persist over time either because this evolution does not affect significantly the viral fitness, or more likely because evolution is constrained through the acquisition of compensatory mutations [69]. However there is not yet any clear answer to that question since several recent reports suggested that HIV-1 was able to escape autologous broadly NAbs without any reduction in fitness [70], [71] whereas others suggested that escape mutations resulted in a decreased ability of the virus to replicate *in vivo* [72], [73].

We tried to explore the molecular mechanisms underlying the enhanced neutralization resistance of HIV-1. We did not find any global changes in the viral envelope when we compared lengths and numbers of PNGS of either the entire gp120 sequences or each variable loop. This contrasted with previous observations made by Bunnik *et al.* who found that the increased neutralization resistance of HIV-1 coincided with longer V1 regions and more PNGS in this region [50]. The limited number of samples used in both studies may explain the lack of consensus. Alternatively, the difference might be attributed to the nature of the populations, maybe more restricted in the Amsterdam cohort than in our sample of patients enrolled in the entire country. When focusing on residues or regions targeted by each HuMoNAb, we did not identify any signature pattern in sequences from contemporary patients that could explain their increased resistance. Nevertheless, a few substitutions previously described as being associated to resistance were present in several of our resistant viruses. For instance, variants lacking the PNGS residue at position N160 were resistant to PG9, PG16 and PGT145, or variants with a shift of a PNGS from position N332 to N334 were resistant to PGT121 and PGT128 [41], [48], [49], [59], [60]. Although these mutations were present in some viruses, they were not sufficient to explain the observed difference in neutralization sensitivity between viruses from historical and contemporary patients. Thus, some HIV-1 variants were found to be resistant despite having conserved residues at key positions required for neutralization suggesting that other molecular determinants should be involved.

Our study focused on subtype B HIV-1 variants issued from French patients. A phylogenetic analysis of the gp160 sequences of these variants with a large series of gp160 sequences issued from clade B variants isolated at the time of primary infection from patients of various geographic origins, suggested that our variants did not belong to a genetically-restricted subset of viruses, but could be considered as representative of the global evolution of clade B viruses worldwide over the three last decades. Taken together, the first study by Bunnik *et al.* and our independent work, performed in two different countries, suggest that the increased neutralization resistance of subtype B HIV-1 variants over the course of the epidemic is a global phenomenon. We cannot draw any conclusion about the evolution of the neutralization properties of other HIV-1 clades, even if a similar trend can be expected since it can be considered that they have been exposed to similar host selective pressures.

Despite the increased neutralization resistance of HIV-1 over time, four HuMoNAbs, NIH45-46^G54W^, VRC01, PGT121 and PGT128, still displayed an important efficacy against the HIV-1 variants from contemporary patients. They neutralized from 78.6 to 85.7% of variants at concentrations ≤10 µg/mL and from 42.9 to 78.6% at concentrations ≤1 µg/mL, even if the breadth of VRC01 was approximately 2-fold lower than previously reported [39], [59], [60]. In contrast, a lower neutralization breadth was observed for PG9, PG16, PGT145, PGT135 and VRC03, which neutralized only from 21.4 to 57.1% of the contemporary HIV-1 variants at concentrations ≤10 µg/mL and from 7.1 to 42.9% at concentrations ≤1 µg/mL. The neutralization breadth of PG9, PG16 and PGT145 was 4- to 5-fold lower than in previous reports which included viruses from various calendar periods [38], [41], [59], [60]. In a vaccinal perspective, this emphasizes the importance of having an updated panel of circulating viruses to identify the epitope specificities that must be targeted preferentially. We next sought to determine which combination of HuMoNabs would provide the best neutralization coverage toward the contemporary HIV-1 variants with the best potency. An IC_50_ heatmap analysis showed that the engineered antibody NIH45-46^G54W^ was the broadest and most potent antibody. It neutralized all HIV-1 variants except two. These two NIH45-46^G54W^-resistant variants were neutralized potently by PGT128, suggesting that combining NIH45-46^G54W^ with PGT128 would efficiently neutralize most variants. NIH45-46^G54W^ is an engineered mutant derived from a clonal variant of VRC01, whose substitution of a tryptophan for a glycine at position 54 was found to increase its potency to about tenfold [40]. As expected, VRC01, the natural counterpart, showed the same neutralization breadth but a lower potency, suggesting that higher concentrations of a combination of VRC01 with PGT128 would be necessary to achieve similar neutralization. These assumptions were confirmed experimentally using mixtures of NIH45-46^G54W^ and PGT128 and of VRC01 and PGT128, showing the complementary effect of these antibodies. In addition, our experimental data clearly indicate that the two complementary categories of antibodies do not compete by steric hindrance and may be efficient in “real-life” conditions. These results are encouraging since a recent study showed that this combination neutralized 96% of a panel of 45 viruses of diverse subtypes [60]. However, most of the studied variants included in that study were issued from patients who had been infected more than 15 years ago. Recent advances in vector-mediated immunoprophylaxis suggest that this gene therapy-based approach could be a promising strategy to bypass the natural immune response and deliver broadly NAbs, thus conferring a sterilizing protection [8], [11], [74]. In this context, our data support that a NIH45-46^G54W^–PGT128 combination should be included in future human trials of immunoprophylaxis.

In addition, to verify whether the evolution of HIV-1 towards enhanced resistance to neutralization has coincided with a poorer capability to induce NAbs, we compared the neutralizing activity of sera from 60 HIV-1 infected patients enrolled at the two extreme periods of the study (1987–1991; 2003–2007). Again, we took a special attention to select samples from patients that would be as comparable as possible. Serum samples were collected at least 3 years (extremes: 35–52 months) after diagnosis of acute infection from patients of similar transmission group (MSM), infected by a clade B virus, with comparable genetic background (Caucasian), and before introduction of any antiretroviral treatment. This delay was selected based on the facts that NAbs, when present, are usually detected after 2 to 3 years, and that many patients were treated passed this delay. We observed a significant reduction of the neutralizing activity of sera from individuals infected later in the epidemic (2003–2007) (lower frequency/titers of heterologous NAbs and reduced neutralization breadth) when compared to that of sera from patients infected earlier in the epidemic (1987–1991). Although the median time of collection differed by 4.5 months between the 1987–1991 sera and those of 2003–2007 it can be postulated that a so slight difference should not have any impact on the results. As similar observations were reported by Bunnik *et al.* [50], the data suggest collectively that the increasing resistance of the HIV-1 viral species to antibody neutralization would be associated to a lowered immunogenicity.

In conclusion, our results confirm a clear continuous and progressive enhanced resistance of HIV-1 to neutralization over time, providing evidence for an ongoing adaptation of the HIV-1 species to the humoral immunity of the human hosts over the course of the epidemic. However, despite this HIV-1 evolution, we found that one combination of two HuMoNAbs still should neutralize all the most recently circulating clade B variants, even at a relatively low concentration. These data provide a rationale for the selection of the HuMoNAbs that should be preferentially used for HIV immunoprophylaxis. They also suggest that a regular surveillance of the sensitivity to neutralization of the most recent transmitted variants will be necessary in the future, especially if this drift toward an enhanced resistance is also observed for other prevalent HIV-1 clades in other regions of the world.

## Materials and Methods

### Ethics statements

Ethic national committees approvals were obtained for the two cohorts [SEROCO: Commission Nationale de l'Informatique et des Libertés (CNIL); PRIMO: Comité Consultatif de Protection des Personnes dans la Recherche Biomédicale (CCPPRB) Paris-Cochin and Comité de Protection des Personnes (CPP) Ile de France III] and all patients gave written informed consent to participate in the cohort.

### Study population

The evolution of the neutralization sensitivity of variants present at time of HIV-1 primary infection was analyzed on plasma samples collected from historical (HP), intermediate (IP) and contemporary patients (CP) enrolled in two cohorts. HP were enrolled in the ANRS SEROCO CO2 cohort [75] whereas IP and CP were enrolled in the ANRS PRIMO CO6 cohort [76]. In order to be as comparable as possible, samples were selected from MSM, all Caucasian and infected by a subtype B virus. Viruses from 40 patients were studied: 11 infected between 1987 and 1991 (HP), 15 infected between 1996 and 2000 (IP) and 14 between 2006 and 2010 (CP) (Table S1). The estimated date of infection was defined as the onset of symptoms minus 15 days for patients with symptomatic primary infection, or the date of incomplete western blot (presence of antibodies to gp160 and P24) minus 1 month, or the midpoint between a negative and a positive ELISA result for asymptomatic patients [77]–[79].

The evolution of HIV immunogenicity was studied using serum samples from clade B-infected patients enrolled in the same two cohorts. They were infected either between 1987 and 1991 (n = 30; early period) or between 2003 and 2007 (n = 30; late period). All patients were MSM, of Caucasian origin and none of them received an antiretroviral therapy before the date of serum samples used for this study. The selected serum samples were collected at least three years after infection (Table S2). This delay was chosen for two reasons. First, it is usually considered that 3 years are sufficient to detect NAbs. Second, many patients started to be treated after this delay. As shown in Table S2, the delay since infection, the virus load and the CD4+ T-cell count at time of sample collection were similar in both groups of patients.

### Nucleic acid extraction, PCR and cloning

HIV-1 RNA was extracted from plasma using the QIAamp Viral RNA Mini Kit (Qiagen). Full length (gp160) *env* genes were amplified by nested RT-PCR using group M *env*-specific degenerated primers. The outer primers pair was ExtM5 (5′-ATGGCAGGAAGAAGCGGARRC-3′) and ExtM3 (5′-CTTRTAAGTCATTGGTCTTAAA-GGYAG-3′) and the inner primers pair was IntM5XE (5′-AATT*CTCGAG* AATTCAGAAAGAGCAGAAGACAGTGGCAATG-3′) containing *Xho*I (italicized) and *Eco*RI (underlined) sites and IntM3MX (5′-GGCC*ACGCGT* CTAGACTACTTTTTGACCACTTGCCMCCCAT-3′) containing *Mlu*I (italicized) and *Xba*I (underlined) sites. Reverse transcription (RT) was carried out using outer primer ExtM3 and the Superscript III First strand synthesis system (Invitrogen). It was followed by the first round of PCR, using the Platinium PCR SuperMix High Fidelity (Invitrogen) with the following conditions: 2 min at 94°C, then 35 cycles of 15 s at 94°C, 30 s at 55°C and 3 min at 68°C, and a final extension step of 10 min at 68°C. A 5 µl aliquot of the products from the first round of PCR was then used as template for the second round of amplification under the same cycling conditions. The amplification products were libraries of *env* genes that represented the diversity of the viral *env* sequences present in the patient population. Each fragment was approximately 2.6 kb in length, spanning the entire open reading frame of the HIV-1 gp160 polyprotein. PCR amplification products were digested with *Xho*I and *Xba*I or *Xho*I and *Mlu*I restriction enzymes (New England Biolabs), purified by agarose gel electrophoresis, and ligated into *Xho*I and *Xba*I or *Xho*I and *Mlu*I digested pCI mammalian expression vector (Promega). The resulting pCI-*env* plasmids representing the amplified virus populations were propagated by transformation of Electromax DH5α electrocompetent *Escherichia coli* (Invitrogen). Library of pCI-*env* plasmids were purified from transformed cultures using silica column chromatography (Macherey-Nagel).

### *Env*-pseudotyped virus production and titration

Env-pseudotyped viruses were produced as previously described [23] by cotransfecting 3×10^6^ 293T cells with 4 µg of each patient-derived pCI-*env* library or of each pCDNA3.1-*env* clone (reference *env* clones of clade B with moderate - tier 2 - sensitivity to neutralization: QH0692.42, AC10.0.29, RHPA4259.7 or REJO4541.67; NIH AIDS Reagent Program) and 8 µg of pNL4.3.LUC.R_E_[80] using FuGene-6 transfection reagent (Promega). Virus stocks were harvested 72 h later, purified by filtration (0.45 µm filter) and stored as aliquots at −80°C. Viral infectivity was monitored by infection of 1×10^4^ TZM-bl cells, with serial fivefold dilutions of viral supernatants in quadruplicate, in the presence of 30 µg/ml DEAE-dextran. Infection levels were determined after 48 h by measuring the luciferase activity of cell lysates using the Bright-Glo luciferase assay (Promega) and a Centro LB 960 luminometer (Berthold Technologies) [81]. Wells producing relative luminescence units (RLU) >2,5 times the background were scored as positive. The TCID_50_ was calculated as described previously [82].

### HIV-1 primary isolates production and titration

The primary isolates BX08 and 92BR020 were used as reference tier 2 strains [30], [61], [62]. Virus stocks were prepared by passaging isolates once or twice on phytohemagglutinin-stimulated peripheral blood mononuclear cells and stored as aliquots at −80°C. Viral infectivity was monitored as described above for Env-pseudotyped viruses.

### Neutralization assay

Sensitivity to heterologous sera and/or to HuMoNAbs of the pseudotyped viruses and of the primary isolates was assessed in duplicate in TZM-bl cells [83], [84]. After titration, virus stocks were diluted to 400 TCID_50_/mL. Aliquots of 50 µL were then incubated for 1 h at 37°C with 50 µL of either 3-fold serial dilutions of heat inactivated serum samples (1∶20 to 1∶540), b12, 4E10, 2G12, 2F5 (50 µg/ml to 0.022 µg/ml, Polymun Scientific), or PGT121, PGT128, PGT135, PGT145, PG9, PG16, NIH45-46^G54W^, VRC01 and VRC03 (10 µg/ml to 0.0046 µg/ml, IAVI and NIH AIDS Reagent Program). The virus-antibody mixture was then used to infect 10,000 TZM-bl cells in the presence of 30 µg/ml DEAE-dextran. Infection levels were determined after 48 h by measuring the luciferase activities of cell lysates, as described above. Results were expressed as mean values. IC_50_ values were defined as the reciprocal of the serum dilution or the antibody concentration required to reduce RLUs by 50%. For immunogenicity studies, the potency score of each serum was calculated by summing the IC_50_ values obtained for each virus divided by the median IC_50_ value for each virus across all serum samples [85].

### Sequence analysis

All *env* PCR products were sequenced according to the Dye Terminator cycle sequencing protocol (Applied Biosystems, Foster City, Calif.). All sequences have been submitted to GenBank and assigned accession numbers KC699001 to KC699040. Sequence alignments were performed using ClustalW in the software package of BioEdit 7.1.11 and edited manually. Amino acid positions were identified by the use of standard HxB2 numbering. Potential N-linked glycosylation sites (PNGS) were identified using N-Glycosite tool at the HIV database website (http://www.hiv.lanl.gov/content/sequence/GLYCOSITE/glycosite.html). The *env* sequences corresponded to the population-based sequencing for each patient. Extensive studies have previously shown that the viral population present early after infection is highly homogeneous, in most of the cases represented by only one or a few variants [56], [86]. Although the goal of our study was not to analyze in depth the diversity of the viral population in each patient, our careful examination of the sequencing profiles suggested that the population was highly homogeneous in each case.

Phylogenetic analysis was inferred using the Minimum Evolution method [87]. The tree was drawn to scale, with branch lengths in the same units as those of the distances used to infer the phylogenetic tree. The distances were computed using the Maximum Composite Likelihood method [88] and are in the units of the number of base substitutions per site. Evolutionary analyses were conducted in MEGA5 [89]. A total of 200 nucleotide *env* sequences were included in the analysis. Forty sequences were derived from our study and 160 sequences were downloaded from the HIV database website (http://www.hiv.lanl.gov/). These sequences were selected based on the following criteria: they were issued from clade B infected patients at time of primary infection, with a documented year of isolation between 1990 and 2009 and a documented geographical origin (25 were from Europe, 113 from USA, 6 from Australia, 15 from Trinidad and 1 from Zambia). There was only one sequence per patient.

### Statistical analysis

Differences in IC_50_ values, length of envelope regions and numbers of PNGS between viruses from HP (1987–1991), IP (1996–2000) and CP (2006–2010) were evaluated using a non-parametric Jonckheere-Tersptra test for trend. For calculations, viruses with IC_50_ >10 or <0.0045 (for PGT121, PGT128, PGT135, PGT145, PG9, PG16, NIH45-46^G54W^, VRC01 and VRC03), >50 or <0.02 (for b12, 4E10, 2G12, 2F5), <20 or >540 (for sera) were assigned a value of 10 or 0.0015, 50 or 0.0076, and 6.66 or 540, respectively. The evolution of the neutralization coverage of viruses from HP to CP by the pools of sera and the HuMoNAbs was evaluated using a Chi^2^ test for trend. The frequency of detection and the titers of NAbs among sera from patients infected in 1987–1991 compared to those from 2003–2007 were tested by Chi^2^ or ANOVA tests after adjustment for the strain (n = 6 strains), respectively. Finally, the neutralization breadth and the potency score of the two groups of sera were compared using a Wilcoxon signed ranked test.

## Supporting Information

Table S1 Characteristics of historical, intermediate and contemporary patients from whom Env-pseudotyped viruses were generated.
(DOC)

Tables S2 Characteristics of HIV-1 chronically infected patients from whom sera were used for analysis of the neutralizing activity.
(DOC)

Table S3 Sensitivity to neutralization [IC50 titers (µg/mL)] of HIV-1 pseudoviruses from contemporary, intermediate and historical patients to two pools of sera from patients infected chronically by HIV-1 at two calendar periods (1987–1991 and 2003–2007).
(DOC)

Table S4 Sensitivity to neutralization [IC50 titers (µg/mL)] of HIV-1 clade B variants to sera from patients infected by HIV-1 at two calendar periods (1987–1991 and 2003–2007).
(DOC)
