# Supplementary material for: Evidence for a Continuous Drift of the HIV-1 Species towards Higher Resistance to Neutralizing Antibodies over the Course of the Epidemic
Source: PLoS Pathog. 2013 Jul 4;9(7):e1003477. doi: 10.1371/journal.ppat.1003477 (PMC3701719; doi:10.1371/journal.ppat.1003477)
Supplement: Table S1 — Characteristics of historical, intermediate and contemporary patients from whom Env-pseudotyped viruses were generated. (DOC) [file ppat.1003477.s001.doc]

**Table S1**. Characteristics of historical, intermediate and contemporary patients from whom Env-pseudotyped viruses were generated

| Groups | Patients | Estimated date of infection | Mode of transmission | Months after infection | Log_10_ plasma viral load /mL | CD4^+^ T-cell  count /mm^3^ |
| --- | --- | --- | --- | --- | --- | --- |
| Viruses  2006-2010 (CP) | 130230 | 02/20/07 | MSM | 0.8 | 5.5 | 170 |
|  | 330424 | 05/30/08 | MSM | 1.4 | 5.5 | 326 |
|  | 590110 | 03/17/07 | MSM | 2.3 | 5.1 | 538 |
|  | 590111 | 05/09/07 | MSM | 1.7 | 4.5 | 1273 |
|  | 660118 | 11/19/09 | MSM | 2 | 4.1 | 591 |
|  | 750214 | 11/16/08 | MSM | 1.4 | 5.8 | 586 |
|  | 751730 | 02/25/09 | MSM | 3.1 | 4.9 | 557 |
|  | 751734 | 01/31/10 | MSM | 1.3 | 4.1 | 441 |
|  | 770203 | 04/07/09 | MSM | 1.4 | 4.6 | 771 |
|  | 840104 | 06/30/09 | MSM | 1.6 | 5.1 | 540 |
|  | 920414 | 01/16/09 | MSM | 1.1 | 5.9 | 793 |
|  | 940139 | 08/20/08 | MSM | 1.2 | 5.8 | 1306 |
|  | 940140 | 08/26/08 | MSM | 2.4 | 5.3 | 517 |
|  | 940218 | 12/08/06 | MSM | 1.1 | 5.6 | 1277 |
| Viruses  1996-2000 (IP) | 60101 | 11/27/96 | MSM | 2.1 | 5.2 | 1240 |
|  | 60204 | 06/06/99 | MSM | 1.5 | 4.7 | 962 |
|  | 130203 | 07/26/97 | MSM | 1.7 | 5.4 | 209 |
|  | 130206 | 01/17/99 | MSM | 1.1 | 4.7 | 774 |
|  | 310103 | 03/20/00 | MSM | 1.3 | 5.4 | 506 |
|  | 440102 | 04/20/97 | MSM | 1 | 5.4 | 1542 |
|  | 440104 | 04/29/97 | MSM | 1.1 | 5 | 498 |
|  | 750202 | 11/23/98 | MSM | 1.2 | 4.7 | 701 |
|  | 750705 | 11/16/98 | MSM | 1 | 5.1 | 222 |
|  | 750710 | 08/05/99 | MSM | 1.3 | 5.2 | 486 |
|  | 750905 | 03/13/00 | MSM | 0.9 | 5.1 | 461 |
|  | 751002 | 07/04/99 | MSM | 1.1 | 4.3 | 593 |
|  | 751102 | 08/24/97 | MSM | 3.3 | 4.2 | 632 |
|  | 751401 | 09/30/99 | MSM | 1.8 | 5.1 | 619 |
|  | 920203 | 11/06/98 | MSM | 1.3 | 5.3 | 740 |
| Viruses  1987-1991 (HP) | 36 | 11/30/87 | MSM | 2.6 | 4.8 | 507 |
|  | 529 | 05/20/88 | MSM | 6 | 5 | 260 |
|  | 562 | 05/31/88 | MSM | 6.1 | 5.1 | 414 |
|  | 657 | 12/07/88 | MSM | 1.8 | 5 | 605 |
|  | 749 | 01/31/89 | MSM | 1.1 | 5.7 | 1005 |
|  | 757 | 01/13/89 | MSM | 1.8 | 4.2 | 430 |
|  | 819 | 11/30/88 | MSM | 4 | 5 | 360 |
|  | 1058 | 05/17/89 | MSM | 2.7 | 4.5 | 1111 |
|  | 1197 | 09/30/89 | MSM | 1.3 | 5.3 | 546 |
|  | 1639 | 08/11/91 | MSM | 2.2 | 6.1 | 376 |
|  | 1644 | 10/31/91 | MSM | 1.3 | 4.6 | 544 |
