# Supplementary material for: Evidence for a Continuous Drift of the HIV-1 Species towards Higher Resistance to Neutralizing Antibodies over the Course of the Epidemic
Source: PLoS Pathog. 2013 Jul 4;9(7):e1003477. doi: 10.1371/journal.ppat.1003477 (PMC3701719; doi:10.1371/journal.ppat.1003477)
Supplement: Tables S2 — Characteristics of HIV-1 chronically infected patients from whom sera were used for analysis of the neutralizing activity. (DOC) [file ppat.1003477.s002.doc]

**Table S2 : Characteristics of patients from whom sera were used for analysis of the neutralizing activity.**

| Groups | Patients | Estimated date of infection | Mode of transmission | Months after infection | Log_10_ plasma viral load /mL | CD4^+^ T-cell count /mm^3^ |
| --- | --- | --- | --- | --- | --- | --- |
| Sera  2003-2007 | 330227 | 04/16/04 | MSM | 38.1 | 4.76 | 634 |
|  | 330229 | 11/14/04 | MSM | 38.3 | 3.61 | 512 |
|  | 330231 | 07/31/05 | MSM | 45.2 | 4.29 | 532 |
|  | 330409 | 03/07/05 | MSM | 38.8 | 2.33 | 943 |
|  | 340118 | 11/25/04 | MSM | 40.1 | 4.15 | 387 |
|  | 350119 | 05/26/07 | MSM | 36.2 | 4.46 | 534 |
|  | 370203 | 12/15/03 | MSM | 38.6 | 4.14 | 408 |
|  | 440123 | 06/14/04 | MSM | 37.0 | 3.69 | 506 |
|  | 590107 | 07/10/06 | MSM | 50.5 | 4.33 | 330 |
|  | 590110 | 03/17/07 | MSM | 38.6 | 5.13 | 59 |
|  | 660109 | 08/15/05 | MSM | 39.8 | 3.11 | 658 |
|  | 660114 | 06/04/07 | MSM | 39.5 | 4.58 | 660 |
|  | 680115 | 09/02/06 | MSM | 37.7 | 5.68 | 490 |
|  | 680206 | 08/07/06 | MSM | 38.8 | 5.08 | 490 |
|  | 690127 | 09/24/06 | MSM | 38.3 | 4.02 | 590 |
|  | 690218 | 09/08/06 | MSM | 40.5 | 4.54 | 438 |
|  | 750208 | 05/10/04 | MSM | 40.3 | 5.10 | 416 |
|  | 750210 | 08/01/04 | MSM | 44.9 | 5.06 | 542 |
|  | 751109 | 04/25/04 | MSM | 50.7 | 4.61 | 427 |
|  | 751203 | 05/19/04 | MSM | 38.7 | 4.09 | 424 |
|  | 751204 | 12/16/04 | MSM | 37.9 | 4.52 | 583 |
|  | 751313 | 03/21/06 | MSM | 39.0 | 2.32 | 422 |
|  | 751315 | 11/26/06 | MSM | 36.8 | 4.43 | 397 |
|  | 751410 | 03/17/05 | MSM | 38.2 | 5.25 | 712 |
|  | 751420 | 01/23/07 | MSM | 39.0 | 4.22 | 368 |
|  | 751632 | 08/29/04 | MSM | 50.9 | 4.38 | 919 |
|  | 752012 | 06/16/04 | MSM | 45.2 | 5.06 | 327 |
|  | 910112 | 12/26/03 | MSM | 38.0 | 4.28 | 331 |
|  | 920406 | 11/09/03 | MSM | 39.3 | 4.68 | 273 |
|  | 950102 | 03/18/04 | MSM | 39.8 | 3.59 | 319 |
| Sera  1987-1991 | 155 | 07/31/87 | MSM | 45.4 | 4.63 | 468 |
|  | 176 | 09/29/87 | MSM | 51.1 | 4.77 | 590 |
|  | 229 | 10/13/87 | MSM | 44.6 | 4.20 | 379 |
|  | 259 | 12/07/87 | MSM | 42.8 | 4.00 | 530 |
|  | 289 | 11/02/87 | MSM | 43.6 | 4.21 | 382 |
|  | 356 | 03/02/88 | MSM | 42.3 | 3.06 | 581 |
|  | 487 | 03/31/88 | MSM | 43.3 | 5.01 | 456 |
|  | 492 | 07/12/88 | MSM | 41.0 | 4.59 | 614 |
|  | 498 | 02/24/88 | MSM | 51.1 | 3.59 | 348 |
|  | 576 | 08/11/88 | MSM | 42.4 | 4.17 | 370 |
|  | 597 | 08/30/88 | MSM | 41.3 | 3.77 | 510 |
|  | 644 | 10/26/88 | MSM | 39.4 | 2.96 | 428 |
|  | 739 | 08/31/88 | MSM | 42.9 | 3.30 | 514 |
|  | 747 | 11/02/88 | MSM | 41.0 | 3.66 | 578 |
|  | 778 | 03/28/88 | MSM | 51.6 | 4.53 | 375 |
|  | 1016 | 08/31/88 | MSM | 48.0 | 4.72 | 574 |
|  | 1197 | 09/30/89 | MSM | 40.3 | 3.44 | 465 |
|  | 1340 | 07/11/89 | MSM | 44.3 | 4.50 | 345 |
|  | 1376 | 04/15/89 | MSM | 48.5 | 4.32 | 355 |
|  | 1420 | 12/20/89 | MSM | 46.9 | 4.47 | 544 |
|  | 1423 | 06/30/89 | MSM | 48.0 | 4.15 | 397 |
|  | 1439 | 01/07/90 | MSM | 42.6 | 5.22 | 516 |
|  | 1450 | 12/12/89 | MSM | 46.0 | 2.91 | 537 |
|  | 1454 | 02/15/90 | MSM | 41.6 | 3.78 | 445 |
|  | 1520 | 03/18/90 | MSM | 45.4 | 2.66 | 571 |
|  | 1543 | 05/31/90 | MSM | 42.6 | 4.14 | 435 |
|  | 1569 | 06/16/90 | MSM | 41.7 | 4.31 | 496 |
|  | 1625 | 12/22/90 | MSM | 34.7 | 4.80 | 428 |
|  | 1630 | 12/31/90 | MSM | 44.6 | 4.90 | 374 |
|  | 1636 | 05/16/91 | MSM | 46.9 | 3.91 | 637 |
