# Supplementary material for: Evidence for a Continuous Drift of the HIV-1 Species towards Higher Resistance to Neutralizing Antibodies over the Course of the Epidemic
Source: PLoS Pathog. 2013 Jul 4;9(7):e1003477. doi: 10.1371/journal.ppat.1003477 (PMC3701719; doi:10.1371/journal.ppat.1003477)
Supplement: Table S3 — Sensitivity to neutralization [IC50 titers (µg/mL)] of HIV-1 pseudoviruses from contemporary, intermediate and historical patients to two pools of sera from patients infected chronically by HIV-1 at two calendar periods (1987–1991 and 2003–2007). (DOC) [file ppat.1003477.s003.doc]

**Table S3.** Sensitivity to neutralization [IC_50_ titers (μg/mL)] of HIV-1 pseudoviruses from contemporary, intermediate and historical patients to two pools of sera from patients infected by HIV-1 at two calendar periods (1987-1991 and 2003-2007)

|  |  | sera 2003-2007 | sera 1987-1991 |
| --- | --- | --- | --- |
| Groups | Patients | IC_50_ values | |
| Viruses  2006-2010 (CP) | 130230 | **65.37** | **117.19** |
|  | 330424 | **47.14** | **34.53** |
|  | 590110 | **41.73** | **35.63** |
|  | 590111 | **21.88** | **20.17** |
|  | 660118 | **82** | **166** |
|  | 750214 | **<20** | **30** |
|  | 751730 | **<20** | **<20** |
|  | 751734 | **32.12** | **24.88** |
|  | 770203 | **45.29** | **99.36** |
|  | 840104 | **<20** | **<20** |
|  | 920414 | **65** | **40** |
|  | 940139 | **135.23** | **164.84** |
|  | 940140 | **60** | **41** |
|  | 940218 | **>540** | **>540** |
| Viruses  1996-2000 (IP) | 60101 | **111.91** | **>540** |
|  | 60204 | **95.93** | **49.05** |
|  | 130203 | **125** | **80** |
|  | 130206 | **75.99** | **31.28** |
|  | 310103 | **124** | **54** |
|  | 440102 | **36** | **24** |
|  | 440104 | **54** | **160** |
|  | 750202 | **47.45** | **24.88** |
|  | 750705 | **41.17** | **173.1** |
|  | 750710 | **<20** | **52.46** |
|  | 750905 | **<20** | **>540** |
|  | 751002 | **440.74** | **>540** |
|  | 751102 | **154.55** | **>540** |
|  | 751401 | **<20** | **39.04** |
|  | 920203 | **<20** | **<20** |
| Viruses  1987-1991 (HP) | 36 | **54.13** | **280.74** |
|  | 529 | **115** | **70** |
|  | 562 | **337.21** | **133.69** |
|  | 657 | **>540** | **175** |
|  | 749 | **102.23** | **>540** |
|  | 757 | **124.38** | **97.27** |
|  | 819 | **47.96** | **138.28** |
|  | 1058 | **45** | **247** |
|  | 1197 | **136.35** | **173.9** |
|  | 1639 | **93** | **93** |
|  | 1644 | **48.43** | **52.63** |

IC_50_ values are color coded : a red box indicates an IC_50_≥ 1:200 dilution, an orange box indicates 1:20 ≤ IC_50_ < 1:200 and a yellow box indicates an IC_50_ < 1:20
