# Supplementary material for: Evidence for a Continuous Drift of the HIV-1 Species towards Higher Resistance to Neutralizing Antibodies over the Course of the Epidemic
Source: PLoS Pathog. 2013 Jul 4;9(7):e1003477. doi: 10.1371/journal.ppat.1003477 (PMC3701719; doi:10.1371/journal.ppat.1003477)
Supplement: Table S4 — Sensitivity to neutralization [IC50 titers (µg/mL)] of HIV-1 clade B variants to sera from patients infected by HIV-1 at two calendar periods (1987–1991 and 2003–2007). (DOC) [file ppat.1003477.s004.doc]

**Table S4.** Sensitivity to neutralization [IC_50_ titers (μg/mL)] of HIV-1 clade B variants to sera from patients infected by HIV-1 at two calendar periods (1987-1991 and 2003-2007)

|  |  | HIV-1 strains | | | | | |
| --- | --- | --- | --- | --- | --- | --- | --- |
|  |  | BX08 | 92BR020 | QH0692.42 | AC10.0.29 | RHPA4259.7 | REJO4541.67 |
| Sera 2003-2007 | 330227 | **<20** | **<20** | **<20** | **<20** | **<20** | **<20** |
|  | 330229 | **<20** | **<20** | **<20** | **<20** | **<20** | **<20** |
|  | 330231 | **<20** | **<20** | **130,47** | **<20** | **<20** | **51,23** |
|  | 330409 | **<20** | **<20** | **59,92** | **<20** | **<20** | **<20** |
|  | 340118 | **<20** | **<20** | **50,82** | **<20** | **<20** | **<20** |
|  | 350119 | **<20** | **<20** | **<20** | **<20** | **<20** | **<20** |
|  | 370203 | **140,79** | **85,94** | **44,76** | **58,14** | **59,96** | **<20** |
|  | 440123 | **<20** | **<20** | **<20** | **<20** | **<20** | **31,22** |
|  | 590107 | **>540** | **179,57** | **51,23** | **108,26** | **310,19** | **28,88** |
|  | 590110 | **<20** | **<20** | **<20** | **<20** | **<20** | **<20** |
|  | 660109 | **<20** | **<20** | **<20** | **<20** | **<20** | **<20** |
|  | 660114 | **35,99** | **<20** | **103,71** | **<20** | **<20** | **<20** |
|  | 680115 | **<20** | **<20** | **45,27** | **76,19** | **90,87** | **43,27** |
|  | 680206 | **72,19** | **49,51** | **<20** | **<20** | **30,03** | **<20** |
|  | 690127 | **<20** | **<20** | **<20** | **<20** | **<20** | **<20** |
|  | 690218 | **<20** | **<20** | **<20** | **130,15** | **<20** | **<20** |
|  | 750208 | **<20** | **<20** | **50,88** | **<20** | **<20** | **93,16** |
|  | 750210 | **<20** | **<20** | **<20** | **47,41** | **<20** | **26,6** |
|  | 751109 | **<20** | **<20** | **<20** | **111,15** | **122,8** | **87,66** |
|  | 751203 | **<20** | **<20** | **<20** | **<20** | **<20** | **<20** |
|  | 751204 | **<20** | **<20** | **<20** | **<20** | **<20** | **<20** |
|  | 751313 | **<20** | **<20** | **85,73** | **<20** | **22,55** | **78,84** |
|  | 751315 | **87,22** | **39,76** | **41,72** | **<20** | **<20** | **<20** |
|  | 751410 | **<20** | **23,39** | **<20** | **<20** | **<20** | **<20** |
|  | 751420 | **<20** | **24,08** | **<20** | **<20** | **<20** | **<20** |
|  | 751632 | **<20** | **<20** | **36,15** | **<20** | **239,47** | **>540** |
|  | 752012 | **<20** | **<20** | **<20** | **<20** | **147,47** | **<20** |
|  | 910112 | **<20** | **<20** | **38,85** | **<20** | **<20** | **<20** |
|  | 920406 | **<20** | **<20** | **25,97** | **<20** | **<20** | **<20** |
|  | 950102 | **40,52** | **<20** | **<20** | **<20** | **<20** | **<20** |
| Sera  1987-1991 | 155 | **>540** | **137,57** | **71,12** | **47,1** | **>540** | **>540** |
|  | 176 | **126,22** | **151,31** | **<20** | **<20** | **<20** | **86,86** |
|  | 229 | **49,77** | **58,47** | **<20** | **47,54** | **<20** | **159,33** |
|  | 259 | **<20** | **<20** | **64,76** | **<20** | **<20** | **<20** |
|  | 289 | **160,86** | **341,95** | **30,14** | **51,47** | **<20** | **<20** |
|  | 356 | **54,36** | **<20** | **37,82** | **25,43** | **<20** | **<20** |
|  | 487 | **<20** | **<20** | **<20** | **<20** | **<20** | **<20** |
|  | 492 | **>540** | **<20** | **>540** | **137,27** | **87,63** | **>540** |
|  | 498 | **38,64** | **<20** | **143,88** | **<20** | **176,18** | **59,25** |
|  | 576 | **<20** | **<20** | **<20** | **<20** | **<20** | **<20** |
|  | 597 | **<20** | **<20** | **<20** | **<20** | **<20** | **<20** |
|  | 644 | **41,65** | **79,87** | **167,82** | **43,74** | **41,1** | **294,89** |
|  | 739 | **115,06** | **39,17** | **221,76** | **>540** | **<20** | **169,14** |
|  | 747 | **<20** | **<20** | **<20** | **<20** | **<20** | **<20** |
|  | 778 | **<20** | **<20** | **<20** | **<20** | **<20** | **<20** |
|  | 1016 | **>540** | **303,11** | **>540** | **126,51** | **>540** | **>540** |
|  | 1197 | **47,78** | **<20** | **114,18** | **46,06** | **98,11** | **276,34** |
|  | 1340 | **148,29** | **<20** | **45,98** | **<20** | **<20** | **<20** |
|  | 1376 | **<20** | **<20** | **<20** | **<20** | **<20** | **<20** |
|  | 1420 | **394,31** | **58,54** | **81,12** | **241,95** | **121,38** | **<20** |
|  | 1423 | **>540** | **217,42** | **172,08** | **<20** | **20,7** | **114,19** |
|  | 1439 | **113,78** | **39,02** | **36,06** | **<20** | **24,58** | **<20** |
|  | 1450 | **<20** | **<20** | **<20** | **<20** | **<20** | **<20** |
|  | 1454 | **<20** | **<20** | **<20** | **<20** | **<20** | **<20** |
|  | 1520 | **51,43** | **<20** | **<20** | **<20** | **<20** | **32,69** |
|  | 1543 | **<20** | **<20** | **<20** | **<20** | **<20** | **<20** |
|  | 1569 | **339,05** | **<20** | **<20** | **<20** | **>540** | **31,73** |
|  | 1625 | **<20** | **<20** | **<20** | **<20** | **<20** | **<20** |
|  | 1630 | **36,51** | **24,61** | **94,97** | **<20** | **<20** | **<20** |
|  | 1636 | **<20** | **<20** | **29,71** | **<20** | **<20** | **<20** |

IC_50_ values are color coded : a red box indicates an IC_50_ ≥ 1:200 dilution, an orange box indicates 1:20 ≤ IC_50_ < 1:200 and a yellow box indicates an IC_50_ < 1:20
